# Gibberellin Disrupts Hormonal Homeostasis and Anther Integrity to Trigger Sex Reversal in Spinach

**DOI:** 10.3390/ijms26199505

**Published:** 2025-09-28

**Authors:** Tengqi Wang, Ehsan Khalid, Haoming Mao, Yihan Tong, Xinyu Xue, Yuru Tang, Lingmin Cai, Ray Ming

**Affiliations:** 1 College of Life Sciences, Fujian Agriculture and Forestry University, Fuzhou 350002, China; 2 Centre for Genomics and Biotechnology, Fujian Provincial Key Laboratory of Haixia Applied Plant Systems Biology, Key Laboratory of Genetics, Fuzhou 350002, China

**Keywords:** GA, stamen carpelization, sex differentiation

## Abstract

Spinach is a dioecious vegetable and an excellent model for investigating plant sex differentiation. Exogenous gibberellin treatment induced sepal hypoplasia and sex reversal, converting 42% of stamens into pistils in male plants. Transcriptome analysis identified 112 male-biased genes enriched in stamen and pollen development, while hormone profiling revealed coordinated changes in GA, cytokinins, auxin, jasmonic acid, and abscisic acid. Functional assays demonstrated that silencing *SpAMS* or *SpPGIP* caused extensive carpelization, and in situ hybridization localized their expression to developing anthers. Dual-luciferase assays confirmed that *SpAMS* directly activates the B-class gene *SpPI*, and genomic mapping placed *SpAMS* in the pseudo-autosomal region of the Y chromosome. These results indicate that GA disrupts hormonal homeostasis and anther wall integrity, while the *SpAMS–SpPI* pathway regulates tapetal development to maintain male identity. Our findings identify *SpAMS* as a key male-promoting factor in spinach and provide a framework for elucidating sex determination mechanisms in dioecious plants.

## 1. Introduction

Sex is a fundamental biological trait. Sex determination is well understood in a few lineages of animals and plants, but remains unknown in the vast majority of dioecious species. Hermaphrodite is ancestral in flowering plants, accounting for about 89% angiosperm species. Approximately 5% of angiosperms are monoecious and 6% are dioecious. Plants lack overt secondary sexual traits; sex is revealed only at floral maturity [1,2,3]. The formation of unisexual organs underpins differentiation, yielding distinct morphologies among male, female, and hermaphrodite flowers [4,5].

In dioecious spinach (*Spinacia oleracea* L., 2 n = 12), sex is governed by an XY system in which the Y-linked Male-Specific Region of the Y chromosome (MSY) drives stamen development [6]. Sex determination in plants is primarily governed by monogenic, digenic, and polygenic models, all of which ultimately influence the expression of floral organ identity genes [7,8,9].

Spinach’s perianth is reduced and lacks petals, and organ specification still conforms to the canonical ABCDE model [10]: A-class genes pattern sepals, B-class (e.g., *SpAP3*/*SpPI*) instruct stamen identity, C-class (*SpAG*) controls stamen/carpel development, D-class (*SpSHP*/*SpSTK*) regulates ovule formation, and E-class (*SpSEP*) cofactors integrate these MADS-box outputs [11,12]. Recent genomics has refined the physical landscape of sex determination. High-density linkage mapping positioned the sex locus to two adjacent intervals on LG4 (66.98–69.72 cM and 75.48–92.96 cM) [13], while independent GWAS narrowed the sex-determining region (SDR) to a 17.42 Mb block on chromosome 4 (92–103 Mb) [14]. Denovo assembly of XX and YY genomes resolved this SDR into a 24.1 Mb Y-linked region (YLR) containing a 10 Mb male-specific Y-differentiated region (YDR) flanked by 14.1 Mb paracentric inversions, juxtaposed with a 13 Mb inversion in X. The YDR, estimated to have diverged ~3 Mya, is repeat-rich and pseudogene-laden, echoing canonical Y-degeneration [15].

The regulatory role of GA in sex differentiation of spinach has garnered increasing attention. Early studies demonstrated that GA application induces masculinization in long-day spinach varieties [16]. Subsequent research revealed an antagonistic interaction between GA_3_ and ethylene, indicating that GA_3_ treatment increases the proportion of female plants—a seemingly feminizing effect that contrasts with the earlier masculinization report—a response correlated with alterations in peroxidase isozyme profiles [17]. Further investigations confirmed that exogenous GA not only enhances the female-to-male ratio but also induces sex reversal, as detectable through the male-specific marker T11A [18]. Most recently, the proposed *SpGAI-SpSTM-SpPI* regulatory module has shed light on the molecular mechanism whereby GA promotes stamen development and facilitates female-to-male sex reversal [19]. A summary of recent studies on GA-induced sex differentiation in spinach reveals inconsistent outcomes regarding the directionality of GA’s effects (Supplementary Table S1, compiled from literature). These discrepancies appear to be associated with variations in experimental conditions, including spinach cultivars, GA concentrations, and treatment timing, underscoring the importance of considering these factors comprehensively in future research.

Functional dissection is now accelerating. *SpMS1*, a pollen-fertility factor conserved across angiosperms, is indispensable for stamen development; its silencing yields male sterility, and its interaction with *SpAP1* positions it at the nexus of organ initiation and sex differentiation [20]. Conversely, B-class genes—exemplified by *SpPI*—display strict male-specific activation; their absence in females defines a clear transcriptional boundary between sexes and suggests that suppression of *B-class* expression is a downstream effect of the Y-linked sex-determining cascade [12,21]. Together, the delimited SDR, sex-linked markers (T11A, SpoX, KASP) [22,23], and gene-level interrogations provide a cohesive genomic and developmental framework for dissecting how MADS-box networks are rewired by an evolving Y chromosome to establish dioecy in spinach.

Integrating transcriptomics, endogenous hormone profiling, and VIGS-based functional assays, this study provides initial evidence that exogenous GA may facilitate the pistil-to-stamen transition in *Spinacia oleracea*, offering new experimental insights into the role of GA in plant sex determination. Moreover, quantifying multiple endogenous hormone levels under GA treatment reveals hormone crosstalk during sex differentiation, offering a fresh perspective on phytohormone-mediated sex control.

## 2. Results

### 2.1. Gibberellin Plays a Dual Role in Regulating Spinach Flower Phenotype

**Figure 1:**
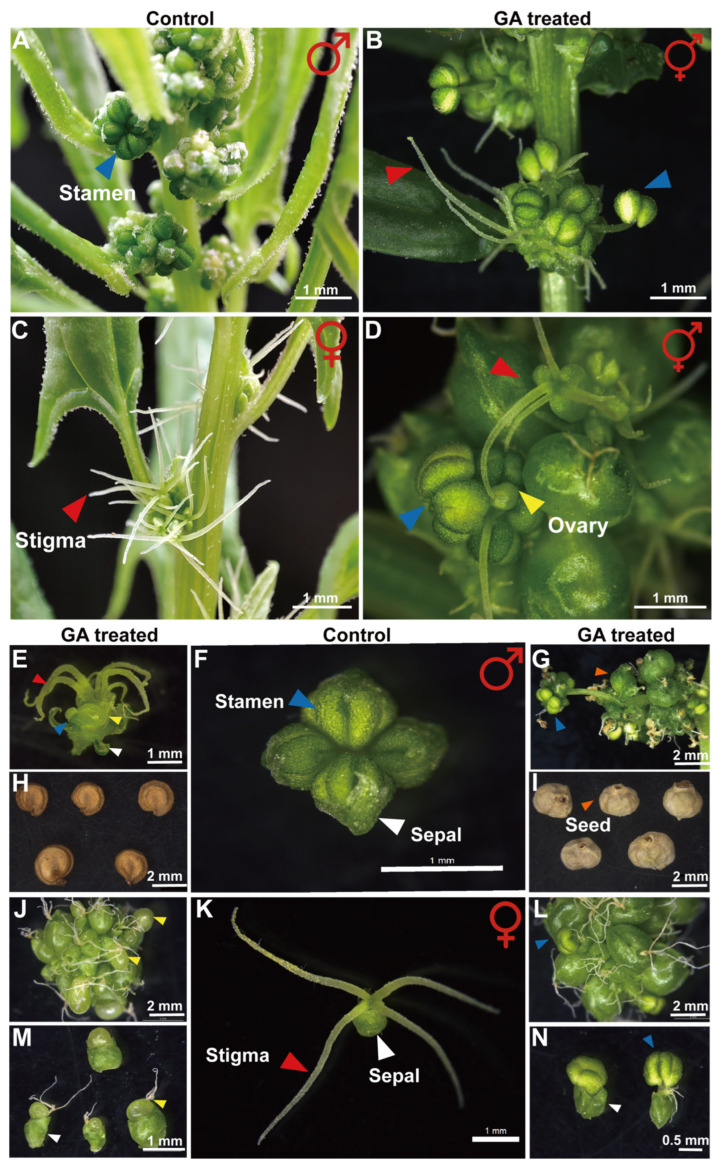
Exogenous GA impact on sexual differentiation in Spinach: (**A**) Male spinach plant grown under natural conditions. (**B**) GA-induced ectopic carpel formation in staminate flowers of spinach. (**C**) A female spinach plant grown under natural conditions. (**D**) GA-induced stamen conversion in pistillate spinach. (**E**) GA-induced solitary male flower exhibiting carpeloidy. (**F**) Solitary staminate flower of Spinach under natural conditions. (**G**,**I**) GA-induced carpelized male Spinach produces seeds enclosed by persistent sepals. (**H**) GA-induced carpelized male Spinach yield seedlings lacking sepals. (**J**,**M**) GA-induced floral malformations in female spinach manifested as partially deformed sepals. (**K**) Solitary pistillate flower of Spinach under natural conditions. (**L**,**N**) In GA-treated female spinach plants that underwent masculinization, the resultant stamens exhibited morphological anomalies, including deformed sepals and the production of a single anther. Red arrows indicate stigmata, blue arrows indicate stamens, and yellow arrows indicate ovules in spinach flowers. Orange arrows denote spinach seeds; white arrows denote sepals.

Spinach is a dioecious plant, exhibiting pronounced sexual dimorphism with distinct male and female structures [24]. In pilot assays, 18.6 mg L^−1^ GA_3_ induced partial sex reversal in both sexes, yielding hermaphroditic flowers (Figure 1A–N). We therefore adopted this concentration for repeated foliar applications, which shifted the female/male flower ratio to approximately 1:2. To dissect the GA-driven stamen-to-carpel transition, male seedlings were identified at the two-leaf stage using sex-linked markers, and 50 uniform individuals were selected for treatment; approximately 42% of these plants subsequently exhibited stamen-to-carpel conversion (Supplementary Figure S1A).

At this concentration, GA promoted carpel formation in the fourth whorl of male flowers, with visible stigma structures (Figure 1B), and induced stamen development in female flowers (Figure 1D). The anthers that developed on female plants were subjected to Alexander staining, revealing that the pollen grains were viable and appeared purplish-red (Supplementary Figure S1B). In male plants, GA-induced carpelization exhibited a distinct spatial gradient, with increased stigma formation towards the apex. Within these apical regions, stamens were completely transformed into mature and functional carpels, closely resembling those of normal female plants (Supplementary Figure S1C). This observation suggests that GA induces a progressive sex reversal in male flowers, with the extent of reversal increasing gradually from the middle to the upper floral nodes.

Notably, carpelized flowers produced viable offspring with significant phenotypic variation, exhibiting two distinct phenotypes associated with sepal development: the presence or absence of sepals (Figure 1E–I). During the masculinization of female flowers, functional anthers were enclosed by malformed sepals (Figure 1J–N). This effect became more pronounced with increasing GA concentrations, particularly in female plants treated with 1000 mg/L GA, where the majority of the progeny lacked sepals. Whether sepal development is associated with sex remains to be further investigated.

### 2.2. Transcriptome Analysis and Endogenous Hormone Measurement to Identify Differentially Expressed Genes Associated with Stamen-to-Carpel Conversion in Dioecious Spinach

**Figure 2:**
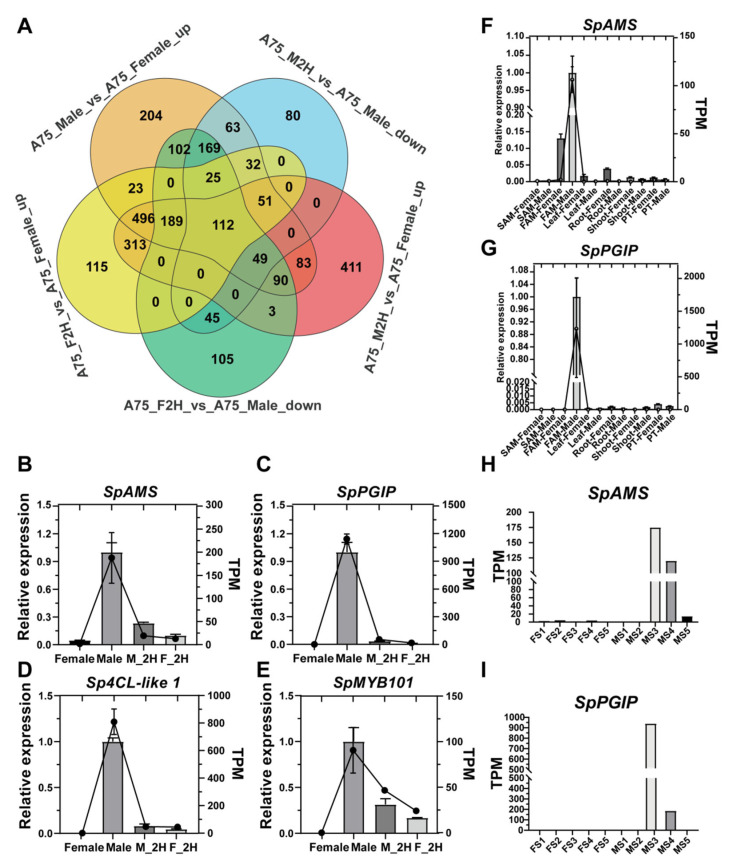
**Transcriptomic analysis and Validation in Spinach:** (**A**) Venn diagram showing the overlap of DEGs among various comparison groups in the spinach transcriptome dataset, revealing a total of 112 candidate DEGs. (**B**–**E**) qRT-PCR was employed to measure the relative expression levels of candidate differentially expressed genes in spinach using three biological replicates, each with three technical replicates. Bar charts show mean ± SD from qRT-PCR quantification, and line charts depict RNA-seq data for *SpAMS*, *SpPGIP*, *SpMYB101* (YY36881) and *Sp4CL-like 1*. (**F**–**I**) Expression Profiles of SpAMS and SpPGIP analyzed by qRT-PCR across different tissues and stages of floral development in spinach. Data points represent mean ± SD from three biological replicates. SAM, stem apical meristem; FAM, flower apical meristem; PT, phase transition; FS1–FS5, five developmental stages of female flowers; MS1–MS5, five developmental stages of male flowers.

To elucidate the genetic basis of GA-induced stamen-to-carpel conversion in spinach, we aimed to identify genes that are highly expressed in male flowers, downregulated during pistil development, and upregulated during stamen formation. Differential expression analysis was performed using criteria of |log2FoldChange| > 2 and *p* < 0.05 across five pairwise comparisons: F_2H (indicating pistil carpelization in female spinach (A75) following GA treatment) vs. Male (1590 DEGs), M_2H (indicating carpeloidy in male spinach following GA treatment) vs. Female (2039 DEGs), M_2H vs. Male (1532 DEGs), Male vs. Female (2157 DEGs), and F_2H vs. Female (1637 DEGs). An integrated analysis of these comparisons–Male vs. Female up, M_2H vs. Male down, M_2H vs. Female up, F_2H vs. Male down, and F_2H vs. Female up–identified 112 candidate genes potentially involved in GA-mediated floral organ transformation (Figure 2A, Supplementary Figure S3, and Supplementary Table S3). Kyoto Encyclopedia of Genes and Genomes (KEGG) analysis highlighted significant enrichment in secondary metabolite biosynthesis pathways (Supplementary Figure S4A and Table S5). Gene Ontology (GO) enrichment analyses revealed that these genes were primarily associated with flower development, anatomical structure morphogenesis, pollen wall formation, gametophyte development, and extracellular structure organization (Supplementary Figure S4B and Table S6).

To elucidate the hormonal regulation underlying GA-induced stamen-to-carpel conversion in spinach, we performed a comprehensive analysis of hormone levels in GA-treated male flowers (M_2H), alongside untreated male and female plants (Supplementary Figure S5).

#### 2.2.1. Specific Alterations in Gibberellin and Jasmonate Profiles in Feminized Flowers

Gibberellin profiling revealed that the GA-induced feminization of male flowers was associated with a significant accumulation of specific gibberellins. The content of GA_29_ in feminized floral organs (M_2H) reached 321.54 ng/g FW, which was 4.0-fold higher than that in untreated female flowers (79.89 ng/g FW). More strikingly, GA_3_ and GA_8_, which were undetectable in both control female and male plants, were specifically induced to substantial levels (45.64 and 4.74 ng/g FW, respectively) in the M_2H group (Supplementary Figure S5D). Similarly, the GA-induced feminization was closely associated with the activation of the JA signaling pathway. In feminized flowers (M_2H), the JA content (428.94 ng/g FW) was 7.5-fold higher than that in untreated male flowers (57.12 ng/g FW). The accumulation of its bioactive form, jasmonoyl-L-isoleucine (JA-Ile), was even more pronounced (504.72 ng/g FW), being 6.9-fold and 3.1-fold greater than the levels in untreated male (72.88 ng/g FW) and female (163.76 ng/g FW) flowers, respectively. Concurrently, the JA biosynthesis precursor, cis-(+)-12-oxophytodienoic acid (OPDA), showed a significant 15.1-fold increase in the M_2H group (249.58 ng/g FW) compared to untreated male flowers (16.53 ng/g FW). Notably, the intermediate metabolite 3-oxo-2-[(Z)-pent-2-en-1-yl]cyclopentanehexanoic acid (OPC-6), which was undetectable in untreated male flowers, accumulated substantially in feminized flowers (436.45 ng/g FW), reaching a level 3.9-fold that of untreated female flowers (110.85 ng/g FW). These results suggest that gibberellin application may trigger an “OPDA → JA → JA-Ile” cascade, significantly enhancing the metabolic flux of the jasmonate pathway, thereby driving the stamen-to-carpel transformation (Supplementary Figure S5C).

#### 2.2.2. GA Treatment Synergistically Enhances Cytokinin Metabolism

Endogenous hormone quantification revealed that cytokinin (CK) metabolites were significantly more abundant in untreated female plants than in male plants. The metabolite tZ9G showed a 14.4-fold increase, and tZ9G-5′MP exhibited an approximately 1-fold elevation in females, indicating that high CK levels are positively associated with female differentiation. Following GA treatment, the CK pool in male plants expanded dramatically: tZ9G levels rose from 18.6 to 473.3 ng/g FW (a 25.4-fold increase), reaching 1.8-fold of the level in untreated females, while tZ9G-5′MP increased from 15.6 to 62.1 ng/g FW (a 4.0-fold rise), attaining 4.3-fold of the female value. These results indicate that GA treatment did not reduce CK contents but rather significantly upregulated both metabolites, suggesting that GA may promote sex reversal by enhancing, rather than antagonizing, CK signaling. This GA-CK synergistic effect provides a novel perspective on the hormonal network underlying pistil development in spinach. However, further investigation, such as blocking CK biosynthesis or attenuating CK signaling, is required to establish causal relationships and determine whether GA-induced feminization is consequently suppressed (Supplementary Figure S5A).

#### 2.2.3. Activation of Auxin and Abscisic Acid Pathways and Suppression of Ethylene Biosynthesis

Furthermore, the levels of auxin and its derivatives were elevated in the floral organs exhibiting stamen-carpel transformation. Quantitative analysis revealed a significant accumulation of these compounds in the M_2H group. Specifically, the indole content in M_2H (3982.34 ng/g FW (fresh weight)) was markedly increased, being approximately 3.5-fold higher than in untreated female flowers (1152.42 ng/g FW) and 2.3-fold higher than in untreated male flowers (1744.74 ng/g FW). The concentration of 3-indoleacrylic acid (IA) in the M_2H group (7.20 ng/g FW) was comparable to that in untreated female flowers (7.40 ng/g FW) but was 2.7-fold higher than in untreated male flowers (2.67 ng/g FW). Notably, 1-O-indol-3-ylacetylglucose (IAA-Glc) was specifically induced by GA treatment; it was exclusively detected in the M_2H group (183.51 ng/g FW) but remained below the detection limit in both untreated female and male flowers. These findings demonstrate that the GA-mediated feminization process is closely associated with a significant accumulation—and, in the case of IAA-Glc, likely de novo synthesis—of specific indole-related compounds, suggesting their potential role as key regulators in sex differentiation (Supplementary Figure S5B). The ABA content in M_2H (27.98 ng/g FW) was significantly elevated, being approximately 2.9-fold and 5.9-fold higher than in untreated female (9.78 ng/g FW) and male (4.77 ng/g FW) flowers, respectively. Notably, its immediate precursor, abscisic aldehyde (ABA-ald), which was undetectable in both untreated female and male flowers, accumulated to a high level (856.95 ng/g FW) specifically in the M_2H group (Supplementary Figure S5F), indicating a specific activation of the ABA biosynthetic pathway by GA. In contrast, the level of the ethylene precursor 1-aminocyclopropanecarboxylic acid (ACC) was markedly suppressed. ACC was undetectable in the M_2H group, whereas considerable levels were found in untreated female (10.42 ng/g FW) and male (12.95 ng/g FW) flowers (Supplementary Figure S5E), suggesting that GA treatment strongly inhibits ethylene biosynthesis.

Collectively, these findings indicate that the stamen-to-carpel conversion in spinach is regulated by a complex interplay of multiple hormones, including GAs, JAs, CKs, IAA, and ABA. The distinct hormone accumulation patterns in M_2H compared to untreated male and female plants underscore the multifaceted hormonal regulation involved in this process. Future research should focus on elucidating the specific interactions between these hormones and their precise roles in spinach sex determination.

Based on the comprehensive analysis, three differentially expressed genes were selected for further experimental validation. The three genes are, respectively, *SpAMS* (YY01740), *SpPGIP* (YY37065) and *Sp4CL-Like 1* (YY28739). Using *PlantCARE* [25] (https://bioinformatics.psb.ugent.be/webtools/plantcare/html/) (accessed on 24 July 2024), we analyzed the promoter regions of the three candidate genes and found that they all contain numerous hormone-responsive cis-acting elements (Supplementary Figure S6), indicating these genes may be regulated by phytohormones. Expression patterns verified by qRT-PCR were consistent with the transcriptome data (Figure 2B–E). Transcriptome profiling across spinach tissues revealed that the two key genes, *SpAMS* and *SpPGIP*, are highly expressed in male floral meristems (Figure 2F,G). Further analysis of sex-specific floral organs at successive developmental stages showed peak transcript levels during the MS3 and MS4 stages of male flower development (Figure 2H,I), suggesting these two stages may be critical for stamen development in spinach.

#### 2.2.4. Functional Validation of Candidate Genes Through Virus-Induced Gene Silencing (VIGS)

**Figure 3:**
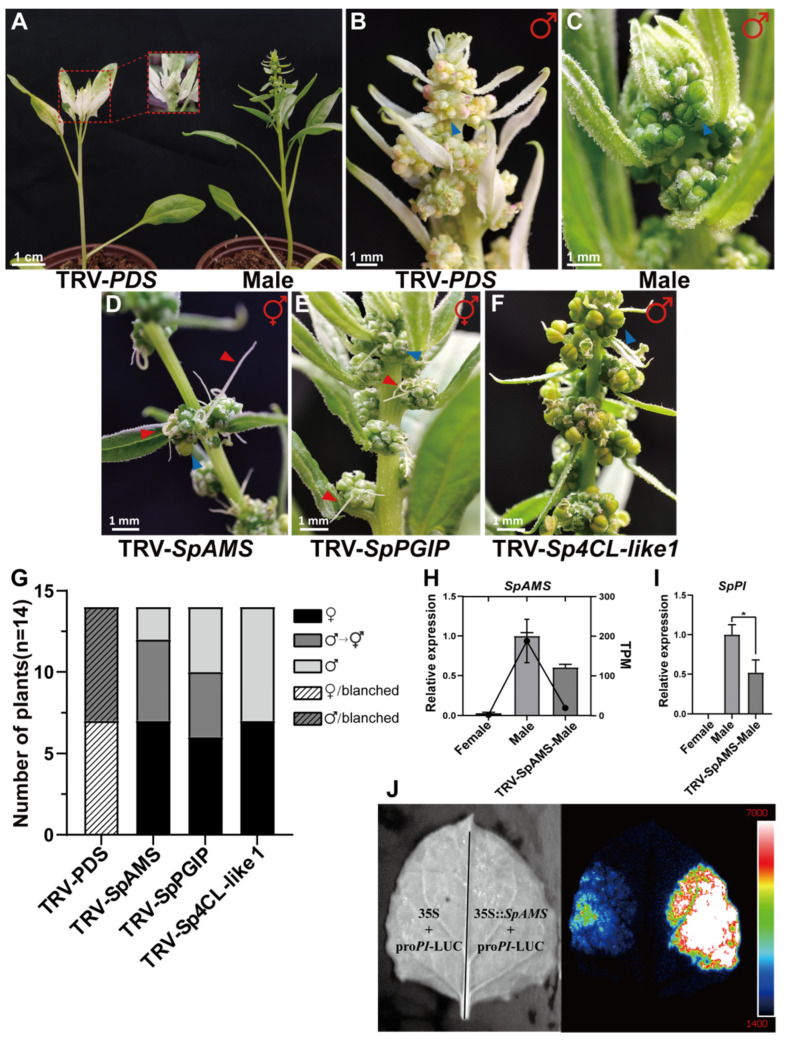
Functional Validation of Candidate Genes by TRV-Induced Gene Silencing (VIGS) in Spinach: (**A**) Male plants with SpPDS silenced and normal male plants. (**B**) Stamens with SpPDS silenced. (**C**) Normal stamens. (**D**) Male plants with carpeloidy after SpAMS silencing. (**E**) Male plants with carpeloidy after SpPGIP silencing. (**F**) Male plants after Sp4CL-Like 1 silencing. (**G**) VIGS plant phenotype transition statistics. Data represent mean ± SD (*n* = 3 biological replicates) from three independent biological replicates. (**H**,**I**) TRV-*SpAMS*-Male: floral organs with carpeloidy in male plants after silencing SpAMS and SpPI genes. Data are presented as mean ± SD (*n* = 3 biological replicates) of three technical replicates. * *p* < 0.05. (**J**) Dual-luciferase assay confirming that the SpAMS transcription factor promotes SpPI expression. Red arrows: Stigma; Blue arrows: Stamen.

To investigate the functions of candidate genes involved in sex differentiation in spinach, we employed the VIGS technique, given the lack of a stable and efficient genetic transformation system for spinach. We targeted three candidate genes: *SpAMS*, *SpPGIP* and *Sp4CL-like 1* (Figure 3D–F). Our results demonstrated that 8 days post-silencing, plants with silenced *SpPDS* (Phytoene Desaturase) exhibited a 100% photobleaching phenotype (Figure 3B,G). The proportions of plants undergoing stamen-to-pistil conversion following silencing of *SpAMS* and *SpPGIP* were 71% and 50%, respectively (Figure 3G). In contrast, silencing *Sp4CL-like 1* had no significant effect. Notably, the phenotypes of *SpAMS*-silenced plants closely resembled those previously reported for *SpPI*-silenced plants (Supplementary Figure S7A,B illustrates the abnormal stamen development observed in *SpAMS*-silenced plants). The sex-converted plants displayed abnormal anthers, characterized by the absence of anthers and the formation of mixed floral organs, suggesting that *SpAMS* and *SpPGIP* may regulate stamen development by suppressing the expression of *SpPI.* As documented in prior studies, *SpPGIP* is crucial for maintaining the integrity of anther primordium cell walls [26], while *SpAMS* is involved in pollen wall formation in the tapetum and serves as a key regulator of pollen development [27]. Transcriptome analysis revealed significantly reduced expression of *SpPGIP* and *SpAMS* in stamens that underwent pistilization (Supplementary Figure S3). Consequently, this may inhibit the expression of *SpAMS*, ultimately interfering with the normal development of the pollen wall and microspores. This cascade of events not only elucidates the potential role of exogenous GA in regulating spinach anther development but also underscores the critical role of the tapetum in spinach sex differentiation. Therefore, we propose that *SpAMS* is one of the key candidate genes regulating sex differentiation in spinach.

Notably, the expression of the male determinant gene *SpPI* was found to decrease following GA application (Figure 3I). To further investigate the regulatory mechanisms, we conducted qRT-PCR analysis on anthers in which the *SpAMS* gene had been silenced. The results revealed that silencing of *SpAMS* led to reduced expression levels of both *SpAMS* and *SpPI* in pollen sacs (tepetum) (Figure 3H). This observation suggests that the carpelization of stamens may be attributed to the downregulation of *SpAMS*. Additionally, bioinformatics analysis using the *JASPAR* database predicted the presence of binding sites for *SpAMS* within the promoter region of the *SpPI* gene (Supplementary Figure S7C). This finding implies a potential regulatory relationship between *SpAMS* and *SpPI*. To validate this hypothesis, we performed dual-luciferase assays in tobacco leaves. The results confirmed that *SpAMS* positively regulates the expression of *SpPI*, thereby supporting its crucial role in male flower development (Figure 3J).

### 2.3. In Situ Localization of SpAMS and SpPGIP Genes

**Figure 4:**
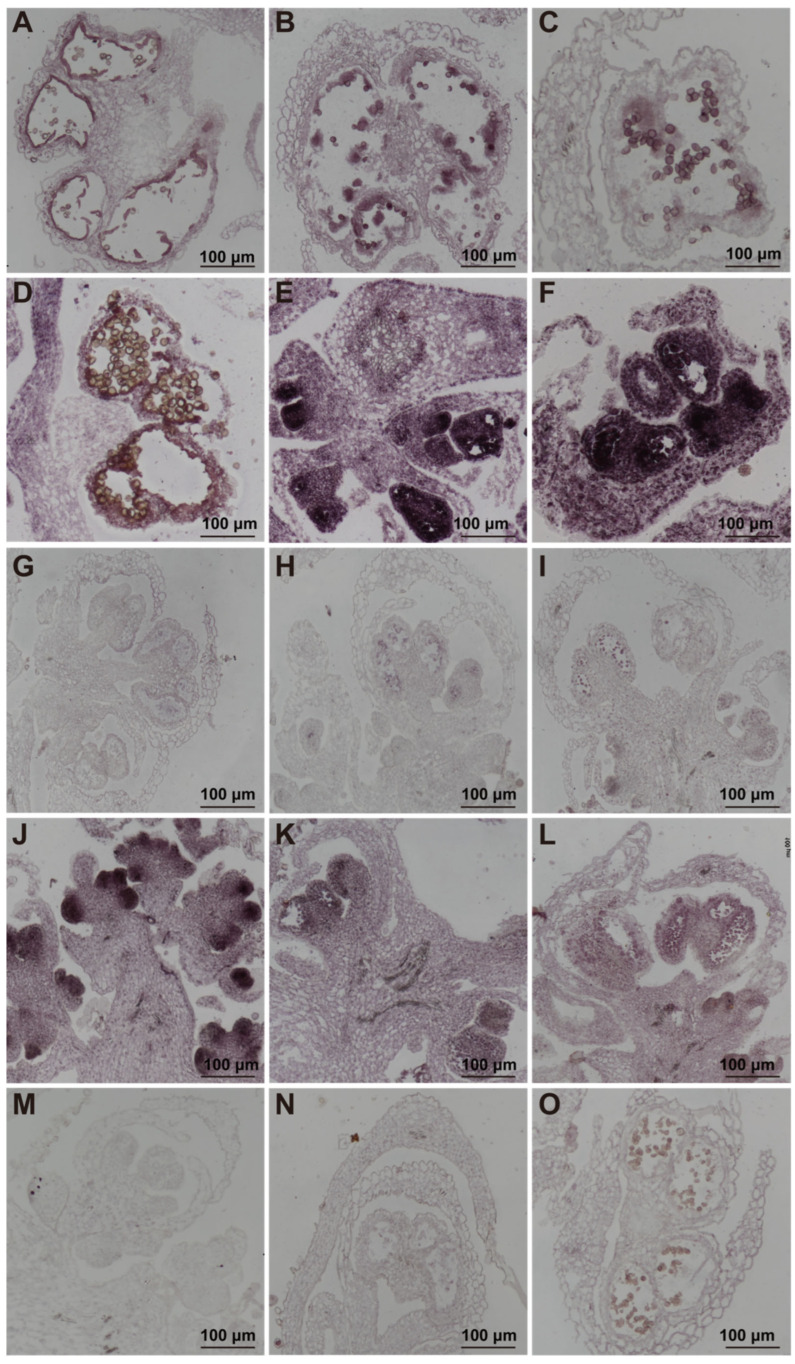
**In Situ Hybridization of spinach *SpAMS* and *SpPGIP* genes during anther development:** (**A**) Displays immature pollen grain stage. (**B**–**D**) Depict the tapetal degeneration stage. (**E**,**F**) Show mature pollen grain stage. (**G**–**I**) Corresponding sense probes for SpAMS. (**J**–**O**) In Situ Hybridization of the spinach SpPGIP. (**J**) Another primordium of the stamen. (**K**,**L**) Mature pollen grain stage. (**M**–**O**) Corresponding sense probes for SpPGIP.

To further elucidate the roles of *SpAMS* and *SpPGIP* in spinach stamen development, we conducted in situ hybridization experiments to determine their expression localization in tissues. Data from the *Arabidopsis eFP Browser* (*TAIR*) (https://www.arabidopsis.org/, accessed on 10 March 2025) revealed that *AtAMS* is highly expressed during mid-stamen development (stages 12–18) in Arabidopsis (Supplementary Figure S9A), highlighting its critical role in tapetum function and pollen maturation [27]. Consistent with this, in situ hybridization results in spinach showed that *SpAMS* transcripts were predominantly localized in the tapetum cells and mature pollen grains (Figure 4A–F), indicating that *SpAMS* plays a significant role in spinach anther development and pollen maturation.

In *Brassica napus*, the expression of the *Bnpgip2-1* gene increases upon pathogen infection but decreases with JA treatment [26]. This is similar to our findings in spinach, where *SpPGIP* expression was significantly reduced in pistillate stamens, while levels of JA and its derivatives were significantly higher compared to control stamens. These results suggest that changes in JA levels may affect the normal expression of the *SpPGIP* gene in spinach stamens (Supplementary Figure S5C). Data from the Arabidopsis eFP Browser showed that *PGIP* is expressed in roots, stems, leaves, and anthers, with particularly high expression in anthers (Supplementary Figure S9B,C). This expression pattern suggests that *PGIP* may be involved in pollen development and protection, contributing to pollen grain formation and maturation and protecting pollen from pathogens. In our in situ hybridization experiments in spinach, *SpPGIP* signals were strongly detected in anther primordia (Figure 4J), similar to the expression pattern in *Arabidopsis*, indicating that the *PGIP* gene plays a key role in anther development. In summary, both *SpAMS* and *SpPGIP* genes are likely closely related to stamen morphogenesis.

### 2.4. Subcellular Localization Analysis of SpAMS and SpPGIP

**Figure 5:**
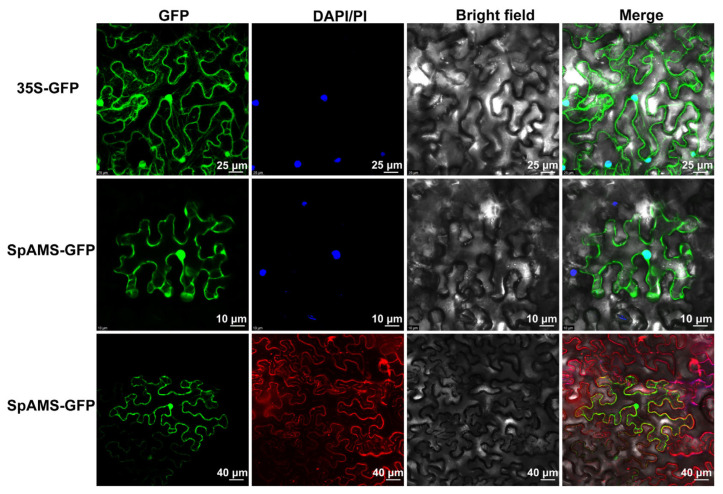
**Subcellular localization of the SpAMS-GFP.** 35S-GFP serves as the blank control. DAPI is used as a nuclear stain. PI is employed as a membrane stain. The second row of four images indicates that SpAMS is localized in the nucleus, while the third row of four images demonstrates that SpAMS is localized in the cell membrane.

To determine its subcellular localization, a p35S::SpAMS-GFP construct was transiently expressed in Nicotiana benthamiana epidermal cells. Confocal microscopy showed SpAMS-GFP localized to both the nucleus and the plasma membrane, indicating dual localization (Figure 5). In contrast, SpPGIP-GFP localized to the cell wall, supporting its role in cell-wall-associated functions (Supplementary Figure S10).

### 2.5. Proposed Regulatory Model of GA Induced Stamen Carpelization in Spinach

**Figure 6:**
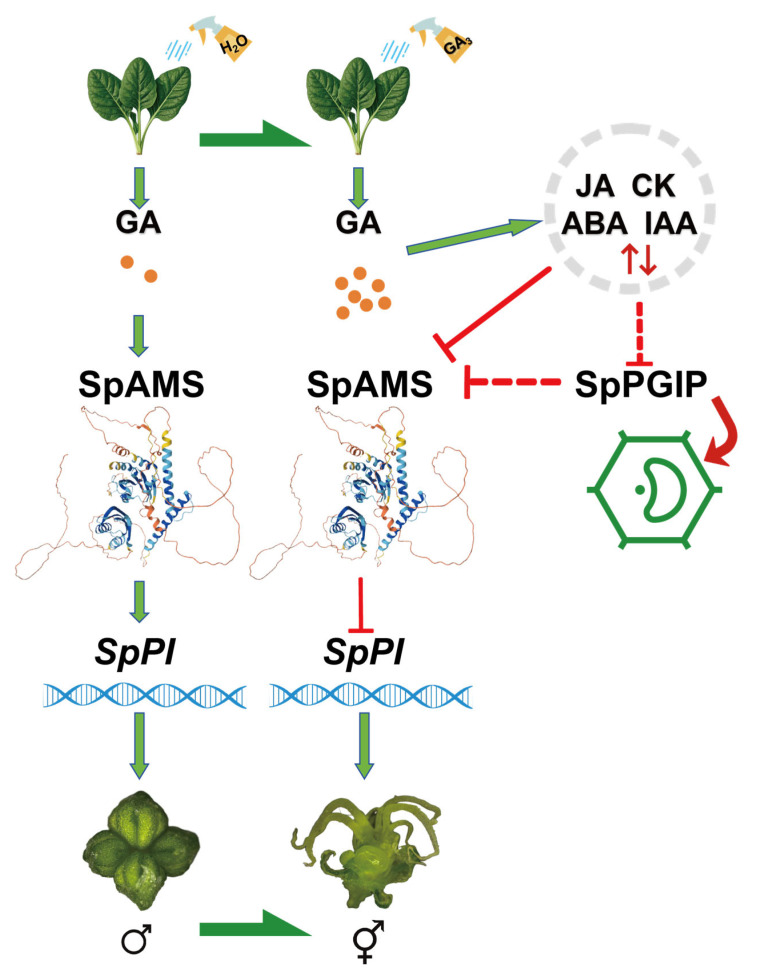
**Regulatory model of GA-induced carpeloidy in spinach.** Green arrows indicate promotion, red targets indicate inhibition, and red dashed-line targets indicate possible inhibition. The model illustrates that under natural conditions, SpPI is normally expressed in spinach anthers. Exogenous GA disrupts endogenous hormone balance, inhibiting the cell-wall factor SpPGIP. This compromises microsporangium cell wall integrity, impairs tapetum development, down-regulates SpAMS, and suppresses SpPI, ultimately disrupting normal anther development.

Based on our experimental findings, we have preliminarily established a regulatory model for GA-induced stamen carpelization in Spinacia oleracea (Figure 6). The application of exogenous GA significantly elevated the endogenous GA levels in S. oleracea and profoundly impacted the contents of other hormones and their derivatives, including JA, CK, ABA, and IAA. These changes in hormone levels likely suppressed the expression of the cell wall-associated gene *SpPGIP* in the anther primordium, thereby compromising cell wall integrity. This disruption subsequently impaired the normal development of the tapetum by downregulating the key tapetum gene *SpAMS*. The process further inhibited the expression of the male-determining gene *SpPI*, thereby disrupting the normal development of stamens and ultimately leading to the emergence of the stamen carpelization phenotype.

## 3. Discussion

This study used dioecious spinach to determine the optimal GA concentration required to achieve complete sex reversal. At the reduced concentration of 18.6 mg·L^−1^, GA exerted bidirectional effects on sex differentiation, corroborating earlier observations [18] that GA can simultaneously activate both masculinizing and feminizing pathways; nevertheless, this concentration was reported to be masculinizing in spinach by Golenberg et al. [28]. We hypothesize that the discrepancy arises from cultivar-specific responses within *Spinacia oleracea*. Statistical analysis revealed a 1:2 ratio of pistillate masculinization to staminate carpelization, indicating a stronger GA-driven feminizing effect on male flowers, consistent with earlier studies [17]. We attribute this discrepancy to the fact that the staminate-carpelization phenotype observed here represents floral re-patterning rather than a true shift in whole-plant sex identity. The GA-driven staminate-carpelization phenotype was predominantly expressed as monoecy, indicating an incomplete sex conversion, whereas only a minority of apical flowers displayed complete female sex reversal. Meta-analysis of recent reports (Supplementary Table S1) underscores the conspicuous variability in GA-induced sex-reversal outcomes, which can be ascribed to cultivar-specific responses, GA dosage, and application regime. Furthermore, both staminate and carpelized flowers induced by GA remained fully fertile. Self-pollination of these flowers yielded F_1_progeny that exhibited variable phenotypes, including seeds either enclosed by or completely lacking sepals. At the highest GA concentrations, seeds were consistently naked. This observation underscores that GA-mediated stamen carpelization does not compromise reproductive viability, paralleling the previous report that cytokinin-treated *Vitis amurensis* produces fertile offspring (Figure 1).

Specifically, exogenous GA establishes a basipetal gradient of floral modification in which stamen-carpelization increases acropetally, mirroring the natural ontogenetic progression of sex differentiation in spinach. By accelerating female organ initiation, GA may extend the reproductive window of male plants and thus enhance their overall fecundity. Spinach is strictly dioecious, and although the master sex-determining gene remains elusive, several loci associated with sex differentiation have been identified [21,28]. Among them, the *DELLA* transcription factor *SpGAI* acts as a female-promoting component within the GA signaling network. Consistent with previous reports, our transcriptomic data revealed significantly higher *SpGAI* expression in female compared with male floral organs [28]. However, in GA-induced carpelized stamens, *SpGAI* transcript levels were indistinguishable from those in untreated male flowers, indicating that additional regulatory factors operate downstream or in parallel to *SpGAI* during stamen carpelization.

In spinach, the B-class *MADS-box* genes *SpAP_3_* and *SpPI* are expressed exclusively during stamen ontogeny and are completely absent from female flowers [12]. Silencing either gene in male plants triggers ectopic pistil formation, underscoring their indispensable role in stamen identity [21,28]. The stamen-to-carpel conversions observed in the present study phenocopy the floral modifications caused by *SpPI* knockdown, suggesting that down-regulation of B-class gene activity underlies the GA-induced phenotype. We propose a model in which GA-induced stamen carpelization is mediated by the downregulation of *SpAMS*, potentially leading to the attenuation of *SpPI* expression, a key determinant of stamen identity. Consistent with this model [19], recently demonstrated that GA regulates male flower development through the *SpGAI–SpSTM–SpPI* module. *SpSTM*, a member of the class-I KNOX family, maintains floral meristem activity and is postulated to interact with genes located within the SDR to influence sexual fate. Here, we identified *SpAMS* as a pseudo-autosomal bHLH transcription factor and propose that it acts as an additional regulator of sex differentiation, potentially through direct or indirect interactions with SDR-linked genes. Exogenous GA application to sweet cherry (*Prunus avium*) markedly elevates endogenous GA and ABA levels and modulates JA and IAA contents. Transcriptomic analyses further revealed that group-III WRKY transcription factors–including *PaWRKY16*, *PaWRKY21*, *PaWRKY38*, *PaWRKY52*, and *PaWRKY53*–are strongly responsive to GA and directly regulate hormone-biosynthetic genes such as *NCED* and *YUCCA* [29]. Metabolomic profiling revealed a synchronized elevation of endogenous GA, CK, IAA, JA, ABA in carpelized stamens (Supplementary Figure S5A–F), elucidating a pivotal mechanism whereby GA rewires the hormonal landscape to override the genetic program of floral sex identity. We demonstrate that exogenous GA triggers a synergistic increase in these endogenous hormones, creating a milieu conducive to feminization. Exogenous GA application induced a significant accumulation of JA and its bioactive conjugates in floral organs, a response consistent with the JA-mediated transcriptional regulation of *BnPGIP2-1* previously reported in Brassica napus [26]. Specifically, *BnPGIP2-1* expression is induced by pathogen challenge but suppressed by JA signaling, suggesting a conserved JA-dependent regulatory mechanism. In spinach, elevated JA levels disrupted the normal expression of *SpPGIP* and *SpAMS* in male floral organs. Concurrently, GA-induced auxin fluctuations suggested a potential role for auxin in floral patterning.

Our transcriptomic analysis identified 112 key differentially expressed genes (DEGs) that are potentially involved in suppressing stamen development and promoting pistil development. Among these candidates, we focused on *SpAMS*, *SpPGIP* and *Sp4CL-Like 1* for functional validation due to their significant differential expression and their putative roles in anther and pollen development based on homologous gene functions in other species. Functional characterization using VIGS demonstrated that silencing *SpAMS* or *SpPGIP* resulted in stamen-to-carpel conversion in 71% and 50% of plants, respectively, highlighting their essential roles in male organ development. In contrast, silencing *Sp4CL-Like 1* produced no discernible phenotypic alterations, indicating it may not play a critical role in this specific process under our experimental conditions. Consistent with prior studies, *SpPGIP* maintains cell wall integrity in anther primordia, while *SpAMS* is involved in pollen wall formation within the tapetum [26,27]. Further mechanistic analysis revealed that *SpAMS* silencing led to the downregulation of the male-determinant gene *SpPI*, and dual-luciferase assays confirmed that *SpAMS* directly activates *SpPI* transcription, establishing *SpAMS* as an upstream regulator of sex determination.

Spatial expression analysis via in situ hybridization localized *SpAMS* transcripts predominantly in tapetal cells and mature pollen grains, whereas *SpPGIP* expression was restricted to anther primordia (Figure 4), consistent with their respective roles in pollen development and primordia integrity.

Based on these findings, we propose a working model (Figure 6) in which GA application elevates JA levels, which in turn suppress *SpAMS* and *SpPGIP* expression, disrupt tapetal differentiation, and ultimately lead to stamen feminization via the interruption of the *SpAMS-SpPI* regulatory module. The direct trans-activation of *SpPI* by *SpAMS* firmly establishes *SpAMS* as a central determinant of spinach sex identity. These results provide a molecular framework for understanding the genetic regulation of reproductive development in spinach and related species.

## 4. Materials and Methods

### 4.1. Plant Materials and Growth Conditions

Spinach germplasm II9A0075 (“A75”) was obtained from the Institute of Crop Sciences, Chinese Academy of Agricultural Sciences, China. Seeds were surface-sterilized with 10% (*v*/*v*) sodium hypochlorite for 5 min and then rinsed three times with sterile deionized water. Sterilized seeds were germinated on absorbent paper-lined Petri dishes in darkness at 22 °C for 5 days [30] (Phcbi MLR-352). Approximately 200 uniformly germinated seedlings were individually transplanted into 10 cm-diameter pots filled with a 1:1.5 (*v*/*v*) mixture of commercial potting soil and vermiculite and cultivated at 22 ± 1 °C under a 16 h light/8 h dark photoperiod in the Fujian Provincial Key Laboratory of Genetics and Genomics, Haixia Applied Plant System Biology Laboratory, China, starting May 2023. Nicotiana benthamiana plants were grown under identical conditions for transient expression assays [31]. A 10 mg mL^−1^ GA stock solution was prepared in 10% (*v*/*v*) ethanol, filter-sterilized (0.22 µm), aliquoted, and stored at −20 °C [28].

### 4.2. Gibberellin Treatment and Tissues Collection

Two hundred spinach plants at the two-true-leaf stage were divided into two groups. The experimental group (*n* = 100) was subjected to foliar spraying with an 18.6 mg/L GA solution [28] (Supplementary Table S1), whereas the control group (*n* = 100) was sprayed with an equivalent volume of deionized water. Treatments were applied every 3 days over 30, yielding a total of 10 applications. Flowering phenotypes—male, female, and hermaphroditic flowers—were monitored and recorded daily. Three types of samples were collected: (i) carpeloid stamens from the experimental group, (ii) pistils, and (iii) stamens from the control group, labeled as M_2H, Female, and Male, respectively. Degenerated floral organs were dissected under a stereomicroscope (Leica DFC550, Wetzlar, Germany), rinsed in 0.9% (*w*/*v*) saline, snap-frozen in liquid nitrogen, and stored at −80 °C (Thermo Scientific^TM^ freezer, Thermo Fisher Scientific (Asheville) LLC, Asheville, NC, USA) for subsequent RNA and hormone analyses.

### 4.3. Molecular Sex Verification

The Y-linked T11A and X-linked SpoX molecular markers were employed for sex verification of male and female two-true-leaf-stage seedlings after GA treatment [22] (Supplementary Figure S2). Primer sequences are listed in Table S2. Genomic DNA was extracted using the cetyltrimethylammonium bromide (CTAB) method [32]. PCR cycling conditions are provided in Supplementary Table S2, and amplicons were resolved on 1.2% (*w*/*v*) agarose gels (Supplementary Figure S2).

### 4.4. RNA Extraction, cDNA Library Construction, Transcriptome Sequencing, and Data Quality Assessment

Total RNA was extracted from spinach floral organs using RNAprep Pure Plant Kit (Tiangen Biotech, Beijing, China). RNA integrity was verified by 1% (*w*/*v*) agarose-gel electrophoresis and quantified with a NanoDrop^TM^ spectrophotometer (Thermo Scientific, Wilmington, DE, USA). Complementary-DNA libraries were prepared with the TruSeq^TM^ Stranded mRNA Library Prep Kit (Illumina, San Diego, CA, USA)and sequenced on an Illumina HiSeq 2500 platform (Illumina, San Diego, CA, USA), generating 150 bp paired-end reads [33]. After quality control with FastQC v0.11.9 and adapter/quality trimming with Trimmomatic v0.39, clean reads were aligned to the spinach reference genome [34] using STAR v2.7.11a. Alignment metrics—mapping rate 89.17% and mismatch rate 0.29%—confirmed high-quality assembly. Transcript abundance was quantified as TPM using StringTie v2.1.7 [35]. Functional annotation was performed with eggNOG-mapper (v5.0) (Available online: http://eggnog-mapper.embl.de/, accessed on 27 August 2024). DEGs were identified using DESeq2 v1.34.0; GO and KEGG pathway enrichment analyses were visualized with the OmicShare platform (https://www.omicshare.com/tools, accessed on 1 August 2024); Supplementary Figure S4A,B).

### 4.5. Endogenous Hormone Profiling

Fresh floral tissues (M_2H, Female and Male) were snap-frozen in liquid nitrogen, lyophilised and analysed by UPLC–ESI-QTRAP 6500+ system (Sciex, Framingham, MA, USA) as detailed in Supplementary Methods. Briefly, 50 mg dry powder was extracted with methanol/water/formic acid (15:4:1, *v*/*v*/*v*) containing deuterated internal standards, filtered (0.22 µm) and quantified by scheduled MRM. Target phytohormones (IAA, CKs, ABA, JA, SA, GAs, ETH, SLs and melatonin) are expressed as ng/g fresh weight. Differential metabolites (|log_2_ FC| ≥ 1, VIP > 1.0 and *p* < 0.05) were identified by OPLS-DA and enriched using KEGG-MSEA (hyper-geometric test). The detailed protocol for Endogenous Hormone Profiling is described in the Supplementary Materials.

### 4.6. Quantitative Reverse Transcription-Polymerase Chain Reaction (qRT-qPCR) Validation

First-strand cDNA was synthesized with 1 µg total RNA using the Evo M-MLV RT Mix Kit with gDNA Clean for qPCR Ver. 2 (Accurate Biotechnology, Changsha, China). After gDNA removal (37 °C for 15 min, 85 °C for 5 s, 4 °C hold), the cDNA was diluted to 200 ng µL^−1^. qRT-PCR was performed with the TB Green Premix Ex Taq II kit (Takara Bio, Kusatsu, Japan) on a LightCycler 480 (Roche, Basel, Switzerland). Gene-specific primers (Table S2) were designed with Primer3Plus. Relative expression was calculated by the 2^−∆∆CT^ method [36] (Supplementary Table S4).

### 4.7. In Situ Hybridization

Gene-specific 300 bp fragments of *SpAMS* and *SpPGIP* were amplified from cDNA and cloned into the *p*TA2 vector (TOYOBO, Osaka, Japan); primer sequences are listed in Supplementary Table S2. Apical male flowers at the early developmental stage (Supplementary Figure S8B,C) were fixed in 4% (*w*/*v*) paraformaldehyde (PFA) at 4 °C for 24 h. After dehydration through an ascending ethanol series (50%, 70%, 85%, 95%, and 100% ethanol, 60 min each), tissues were cleared with xylene (1:3, 1:1, and 3:1 ethanol: xylene, *v*/*v*) and infiltrated with paraffin. Serial 5 µm sections were cut on a rotary microtome (Leica RM2235, Wetzlar, Germany) and mounted on poly-L-lysine-coated slides, which were dried overnight at 37 °C. In situ hybridization was performed as described by Sather [11,21,37]. Slides were washed, dried, and examined under a microscope (OLYMPUS BX53, Tokyo, Japan); images were acquired for further analysis.

### 4.8. Subcellular Localization

The full-length CDS of *SpAMS* (excluding the stop codon) was amplified and cloned into the *SalI* and *SpeI* sites of the *p*2300-GFP vector to generate a C-terminal GFP fusion construct; primers carrying restriction-site adapters are listed in Table S2. The reaction mixture was transformed into Escherichia coli *DH5α*. Plasmids from confirmed clones were extracted and introduced into *Agrobacterium tumefaciens* GV3101 (pSoup-p19) via heat-shock transformation. Transformed *Agrobacterium* was grown in LB medium containing 50 µg mL^−1^ kanamycin and Rifampicin to an OD_600_ ≈ 0.8, harvested by centrifugation (5000× *g*, 10 min), and resuspended in infiltration buffer (10 mM MES, 10 mM MgCl_2_, 200 µM acetosyringone, pH 5.6) to the same OD. After 3 h incubation at 28 °C, suspensions were infiltrated into the abaxial surface of *Nicotiana benthamiana* leaves. Plants were kept in darkness for 24 h at 22 °C, followed by 48 h under a 16 h light/8 h dark photoperiod. Leaf discs (~1 cm^2^) were excised, and GFP fluorescence was visualized using a confocal laser-scanning microscope (Leica TCS SP8, Wetzlar, Germany) [38].

### 4.9. Virus-Induced Gene Silencing (VIGS)

The *p*TRV2 vector was linearized with *EcoRI* and *BamHI*. Gene-specific fragments (~300 bp) of *SpAMS*, *SpPGIP* and *Sp4CL-Like1* were PCR-amplified using primers containing *EcoRI* and *BamHI* extensions and subsequently cloned into the linearized *p*TRV2 backbone via In-Fusion seamless ligation. Agrobacterium-mediated infiltration and plant handling were performed as described in Section 4.8. The infiltration was carried out on the two cotyledons of spinach seedlings at the two-leaf stage (Supplementary Figure S8A).Silencing efficiency in positive plants was evaluated by qRT-PCR; the primer sequences are listed in Supplementary Table S4.

### 4.10. Dual Luciferase In Vivo Imaging Assay

The full-length *SpAMS* CDS was cloned into the effector plasmid *pGreenII 62-SK*, and a 2.0 kb *SpPI* promoter fragment was inserted into the dual-luciferase reporter vector *pGreenII 0800*-LUC using primers harboring appropriate restriction sites (Supplementary Table S2). The method of infection and plant treatment is the same as 4.8. For luciferase imaging, leaves were sprayed with 100 µg mL^−1^ *D-luciferin* (GoldBio, St. Louis, MO, USA), incubated in darkness for 5 min, and imaged for bioluminescence using a NightSHADE LB985 system (Berthold Technologies, Bad Wildbad, Germany) [38].

### 4.11. Statistical Analysis

Statistical analyses were performed using *t*-tests; data are presented as mean ± standard deviation (SD) in bar graphs, and asterisks indicate significant differences (*p* < 0.05, *p* < 0.01). All experiments were conducted with at least three independent biological replicates. Figures were generated using GraphPad Prism 8.3.0 (GraphPad Software, San Diego, CA, USA) and Adobe Illustrator 2024 (Adobe Systems, San José, CA, USA).

## 5. Conclusions

Exogenous GA_3_ at 18.6 mg·L^−1^ triggers a dose-dependent, bidirectional sex conversion in *Spinacia oleracea*: female flowers are masculinized while stamens undergo carpelization; higher concentrations (≥1000 mg·L^−1^) inhibit growth and cause severe floral malformations. This dual effect is mediated by the *SpAMS–SpPI* module, in which GA-induced elevations of JA and other hormones disturb the expression of *SpAMS* and *SpPGIP*, thereby repressing *SpPI* and redirecting stamen fate. Notably, carpelized stamens retain full fertility, and their self-pollinated progeny exhibit sepal-loss phenotypes. Collectively, this study establishes a theoretical framework for GA-mediated sex conversion, identifies the tapetum-critical gene *SpAMS* as a key regulator of sex differentiation, and provides a molecular foundation for further investigations into sex-determining genes in spinach.

## Data Availability

The raw transcriptome sequencing data of spinach (*Spinacia oleracea*) have been deposited in the National Center for Biotechnology Information (NCBI) under BioProject number PRJNA1310857. All data relevant to this study are included in the Supplementary Materials accompanying this paper.

## Supplementary Materials

The following supporting information can be downloaded at: https://www.mdpi.com/article/10.3390/ijms26199505/s1 [39,40,41,42,43,44,45,46,47].
