# Supplementary material for: Gibberellin Disrupts Hormonal Homeostasis and Anther Integrity to Trigger Sex Reversal in Spinach
Source: Int J Mol Sci. 2025 Sep 28;26(19):9505. doi: 10.3390/ijms26199505 (PMC12525398; doi:10.3390/ijms26199505)
Supplement: Supplementary file 1 [file ijms-26-09505-s001.zip › Supplementary Figures.pdf]

Supplementary Text S1

Endogenous Hormone Profiling

Chemicals and reagents

HPLC grade acetonitrile (ACN) and methanol (MeOH) were purchased from Merck (Darmstadt, Germany). MilliQ water (Millipore, Bradford, USA) was used in all experiments. All of the standards were purchased from Olchemim Ltd. (Olomouc, Czech Republic) and isoReag (Shanghai, China). Acetic acid and formic acid were bought from Sigma-Aldrich (St Louis, MO, USA). The stock solutions of standards were prepared at the concentration of 1 mg/mL in MeOH. All stock solutions were stored at -20°C. The stock solutions were diluted with MeOH to working solutions before analysis.

Sample preparatio

Fresh plant sample was harvested, immediately frozen in liquid nitrogen, ground into powder(30 Hz, 1 min), and stored at -80°C until needed. 50 mg of plant sample was weighed into a 2 mL plastic microtube and frozen in liquid nitrogen, dissolved in 1 mL methanol/water/formic acid (15:4:1, V/V/V). 10 μL internal standard mixed solution (100 ng/mL) was added into the extract as internal standards (IS) for the quantication. The mixture was vortexed for 10 minutes, then centrifugation for 5 min (12000 r/min, and 4°C), the supernatant was transferred to clean plastic microtubes, followed by evaporation to dryness and dissolved in 100 μL 80% methanol (V/V), and filtered through a 0.22 μm membrane filter for further LC-MS/MS analysis [39,40].

UPLC conditions

The sample extracts were analyzed using an UPLC-ESI-MS/MS system (UPLC, ExionLC™ AD, https://sciex.com.cn/; MS, QTRAP® 6500+, https://sciex.com.cn/). The analytical conditions were as follows, LC: column, Waters ACQUITY UPLC HSS T3 C18 (100 mm×2.1 mm i.d., 1.8 μm); solvent system, water with 0.04% acetic acid (A), acetonitrile with 0.04% acetic acid (B); gradient program, started at 5% B (0-1 min), increased to 95% B (1-8 min), 95% B (8-9 min), finaly ramped back to 5% B (9.1-12 min); flow rate, 0.35 mL/min; temperature, 40°C; injection volume: 2 μL [41-43].

ESI-MS/MS conditions

Linear ion trap (LIT) and triple quadrupole (QQQ) scans were acquired on a triple quadrupole-linear ion trap mass spectrometer (QTRAP), QTRAP® 6500+ LC-MS/MS System, equipped with an ESI Turbo Ion-Spray interface, operating in both positive and negative ion mode and controlled by Analyst 1.6.3 software (Sciex). The ESI source operation parameters were as follows: ion source, ESI+/-; source temperature 550 ℃; ion spray voltage (IS) 5500 V（Positive）, -4500 V (Negative); curtain gas (CUR) was set at 35 psi, respectively. Phytohormones were analyzed using scheduled multiple reaction monitoring (MRM). Data acquisitions were performed using Analyst 1.6.3 software (Sciex). Multiquant 3.0.3 software (Sciex) was used to quantify all metabolites. Mass spectrometer parameters including the declustering potentials (DP) and collision energies (CE) for individual MRM transitions were done with further DP and CE optimization. A specific set of MRM transitions were monitored for each period according to the metabolites eluted within this period [44-46].

Data processing and statistical analysis

Hierarchical cluster analysis (HCA) was performed with the R package pheatmap (v 1.2.1); metabolite signals were unit-variance-scaled prior to clustering. Differentially accumulated metabolites were selected when $|\log_2 FC| \geq 1$. KEGG annotation and pathway

enrichment were conducted against the KEGG Compound and Pathway databases; significantly enriched pathways were identified by hyper-geometric test ($p < 0.05$) via MSEA.

Pre-treatment of metabolite data

Two scaling approaches were applied:

(1) Unit-variance scaling (UV, auto-scaling): each variable was mean-centered and divided by its standard deviation, yielding a mean of 0 and a standard deviation of 1.

(2) Zero-centering (Ctr): the mean of each variable was subtracted without further scaling.Hierarchical cluster analysis (HCA) was performed with the R package pheatmap (v 1.2.1). Metabolite intensities were unit-variance-scaled prior to clustering. Differentially accumulated metabolites were selected when $|\log_2 FC| \geq 1$. KEGG annotation and enrichment were conducted against the KEGG Compound and Pathway databases; significantly enriched pathways were identified by hyper-geometric test ($p < 0.05$) using MSEA.

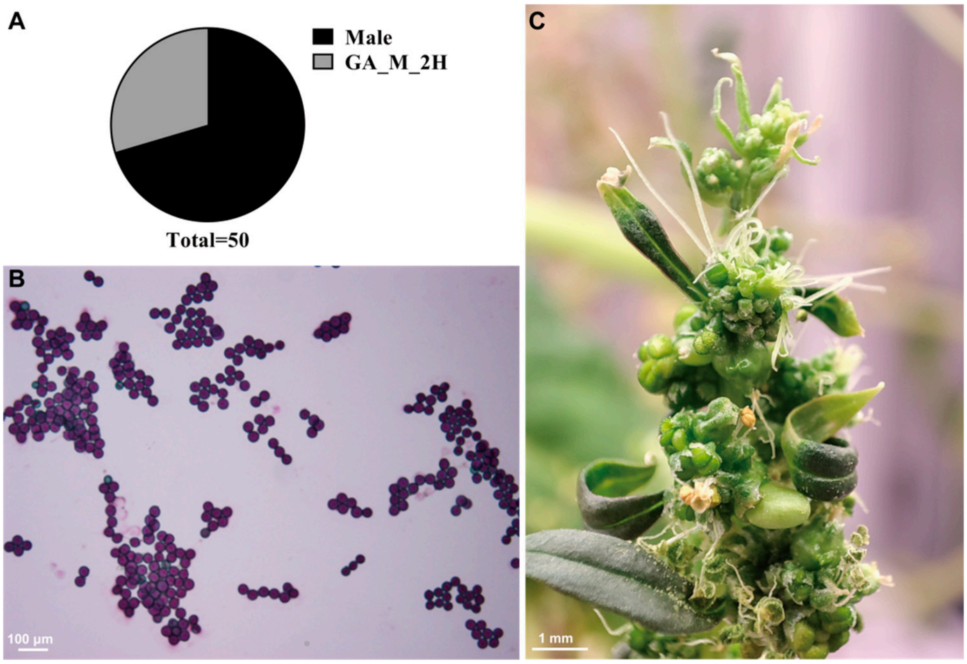

Figure S1. The Impact of Gibberellin on the Sex Determination of Spinach

(A) Phenotypic statistics of gibberellin treatment.Male: total male plants treated with GA (n = 50). GA_M_2H: male plants exhibiting stamen carpelization after GA treatment. (B) Viability of pollen grains from GA-induced stamen carpeloid flowers stained with Alexander's reagent; magenta indicates viable pollen. (C) Spatial gradient of GA-induced stamen-to-carpel conversion in *Spinacia oleracea*. Female characteristics intensify acropetally, with fully converted female flowers localized at the apex.

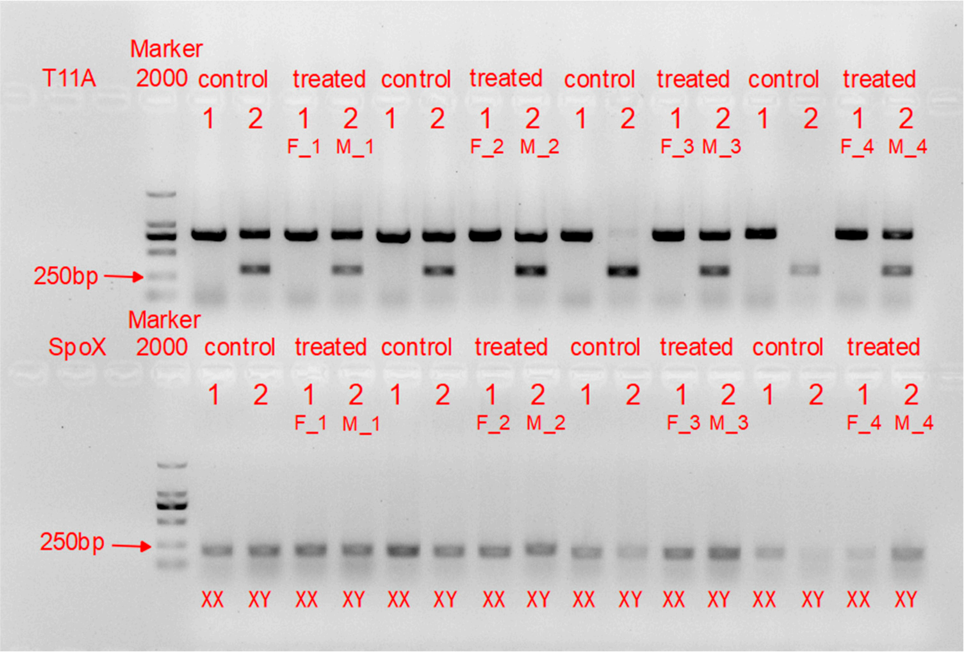

Figure S2. Gender Verification Before and After Gibberellin Treatment.

A 2000 bp DNA ladder served as the size standard. The Y-linked marker *T11A* (320 bp) and the X-linked marker *SpoX* (198 bp) were used. F_1 – F_4: female plants displaying distinct stamen-to-carpel conversion phenotypes. M_1 – M_4: male plants displaying distinct stamen-to-carpel conversion phenotypes. Control: untreated *S. oleracea* plants. Treated: plants treated with 18.6 mg/L GA. Lane 1: Female plants. Lane 2: Male plants. Upper panel: *T11A* amplification; a 320 bp band indicates the presence of the Y chromosome. Lower panel: *SpoX* amplification; a 198 bp band indicates the presence of the X chromosome.

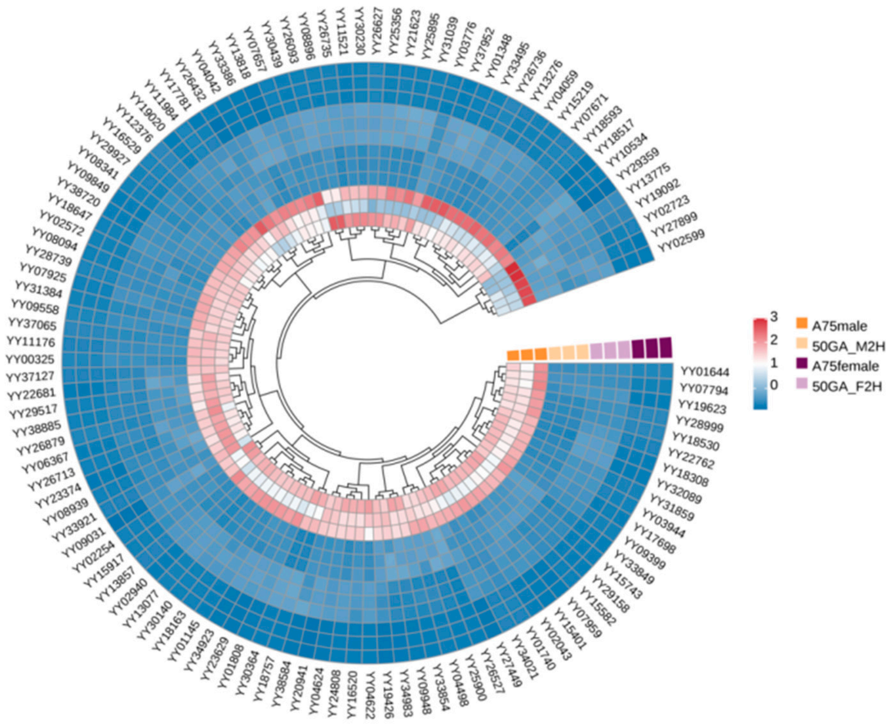

Figure S3. Initial Screening of Transcriptome Identifies 112 Candidate Genes

A75_Male, staminate flowers under natural conditions; 50GA_M2H, hermaphroditic flowers from GA-treated male plants; A75_Female, pistillate flowers under natural conditions; 50GA_F2H, hermaphroditic flowers from GA-treated female plants.

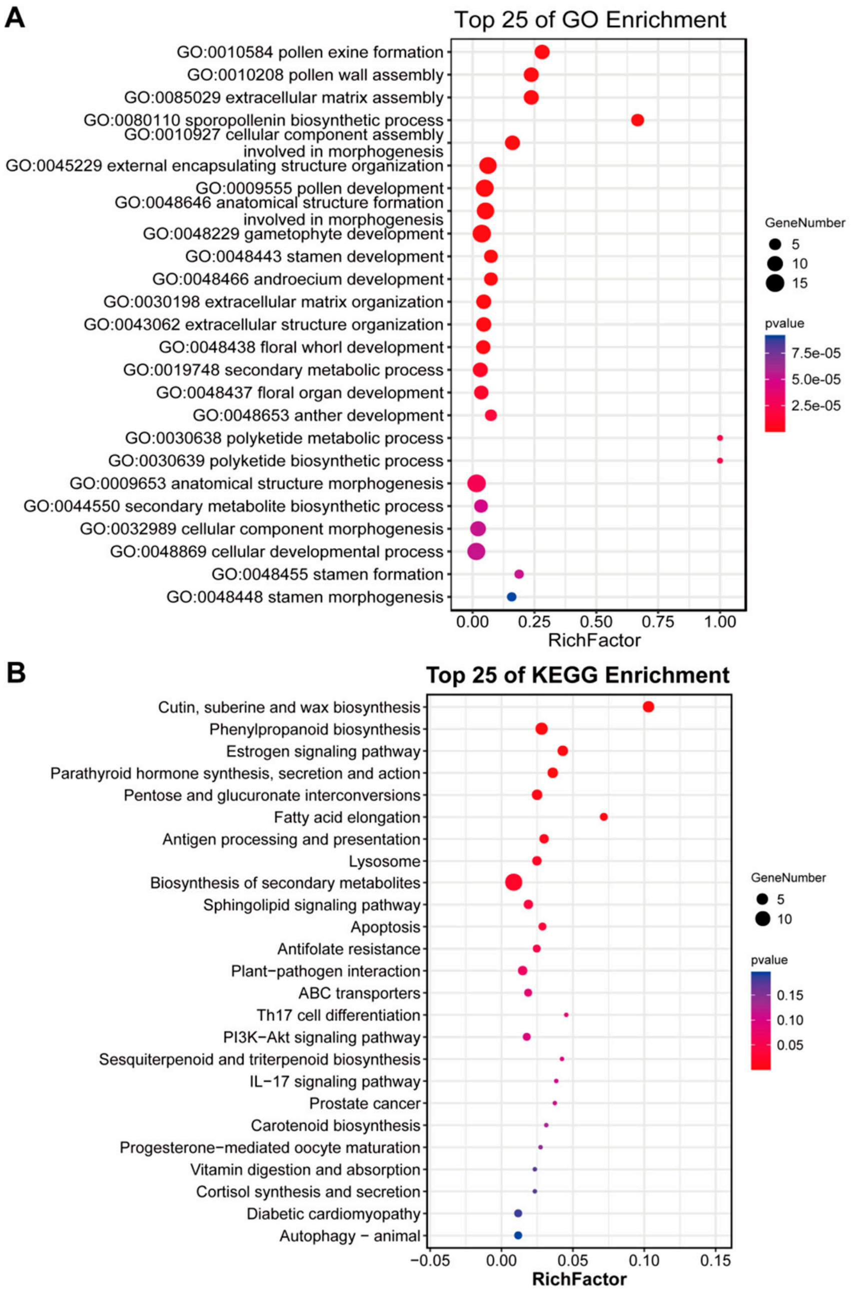

Figure S4. Enrichment analysis of candidate DEGs.

(A) KEGG enrichment analysis of the 112 candidate differentially expressed genes (DEGs). The bubble chart displays the top 25 significantly enriched pathways. The x-axis represents the enrichment factor, the bubble size corresponds to the number of genes, and the bubble color indicates the significance level (red: more significant; blue: less significant). (B) GO enrichment analysis of the 112 candidate DEGs. The bubble chart shows the top 25 significantly enriched GO terms. The x-axis represents the enrichment factor, the bubble size corresponds to the number of genes, and the color indicates the significance level.

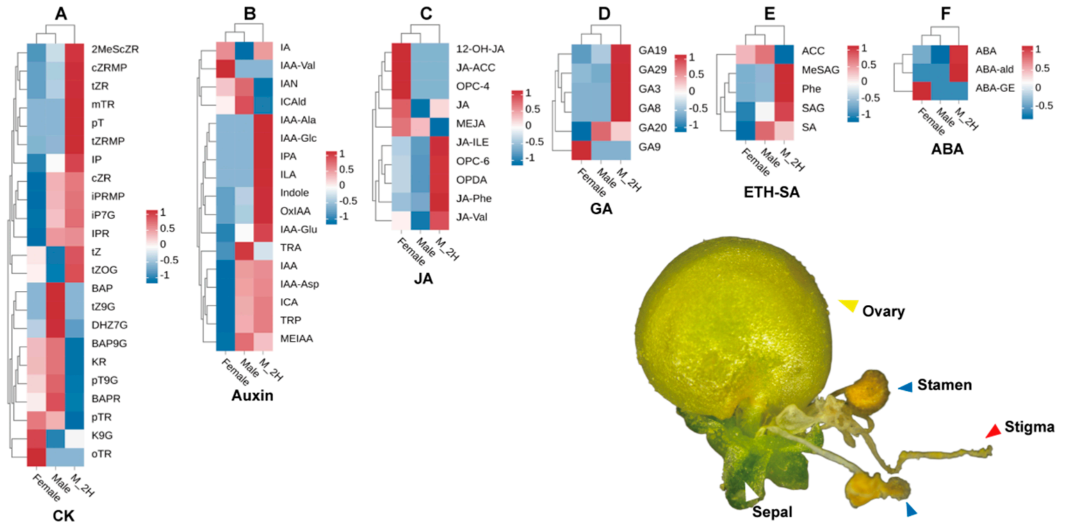

Figure S5. Endogenous Hormone Profiling in Spinach Floral Organs under Control and GA-Treated Conditions

Heat-map analysis of endogenous hormones in Spinacia oleracea floral organs. A75_Female, pistillate flowers from untreated plants; A75_Male, staminate flowers from untreated plants; GAM_2H, carpeloid stamens from GA-treated male plants (three biological replicates per sample) (A) Cytokinins (CKs) and derivatives. (B) Auxin (IAA) and derivatives. (C) Jasmonic acid (JA) and derivatives. (D) Gibberellins (GAs) and derivatives. (E) Ethylene (ETH) and salicylic acid (SA) derivatives. (F) Abscisic acid (ABA) and derivatives. Red boxes indicate hormone contents that were significantly higher in GA-induced carpeloid stamens, whereas green boxes denote significant decreases compared with untreated controls ($p < 0.05$). Symbols: yellow arrow, ovary; red arrow, stigma; blue arrow, stamen; green arrow, sepal.

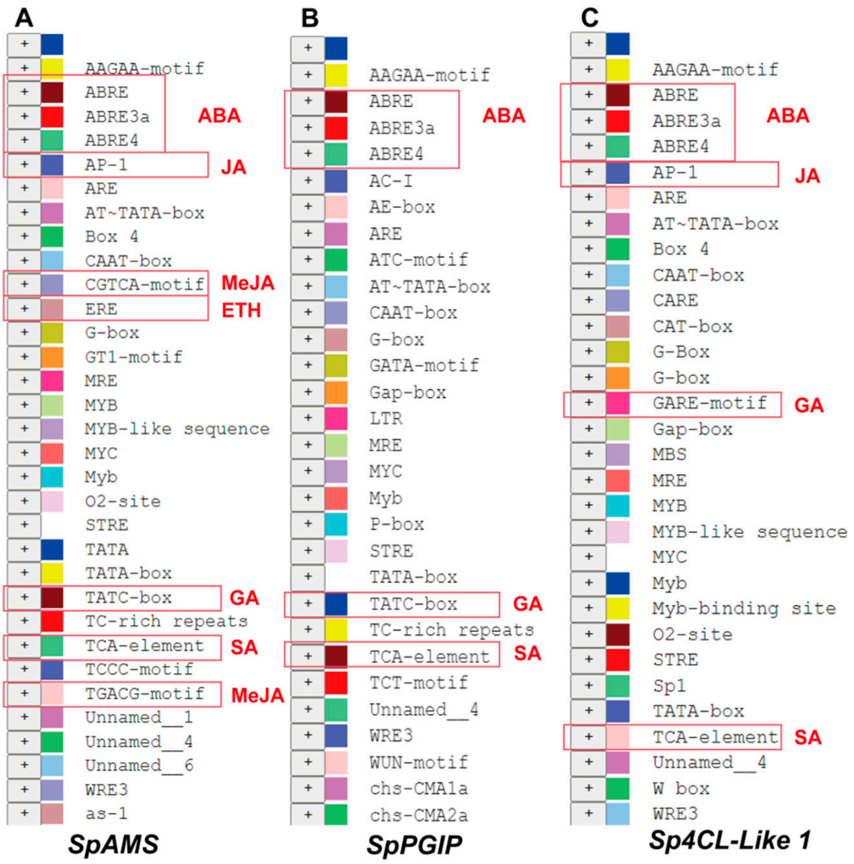

Figure S6. Promoter Analysis of Four Candidate Genes

Red boxes indicate hormone-related cis-acting elements in the promoter regions.

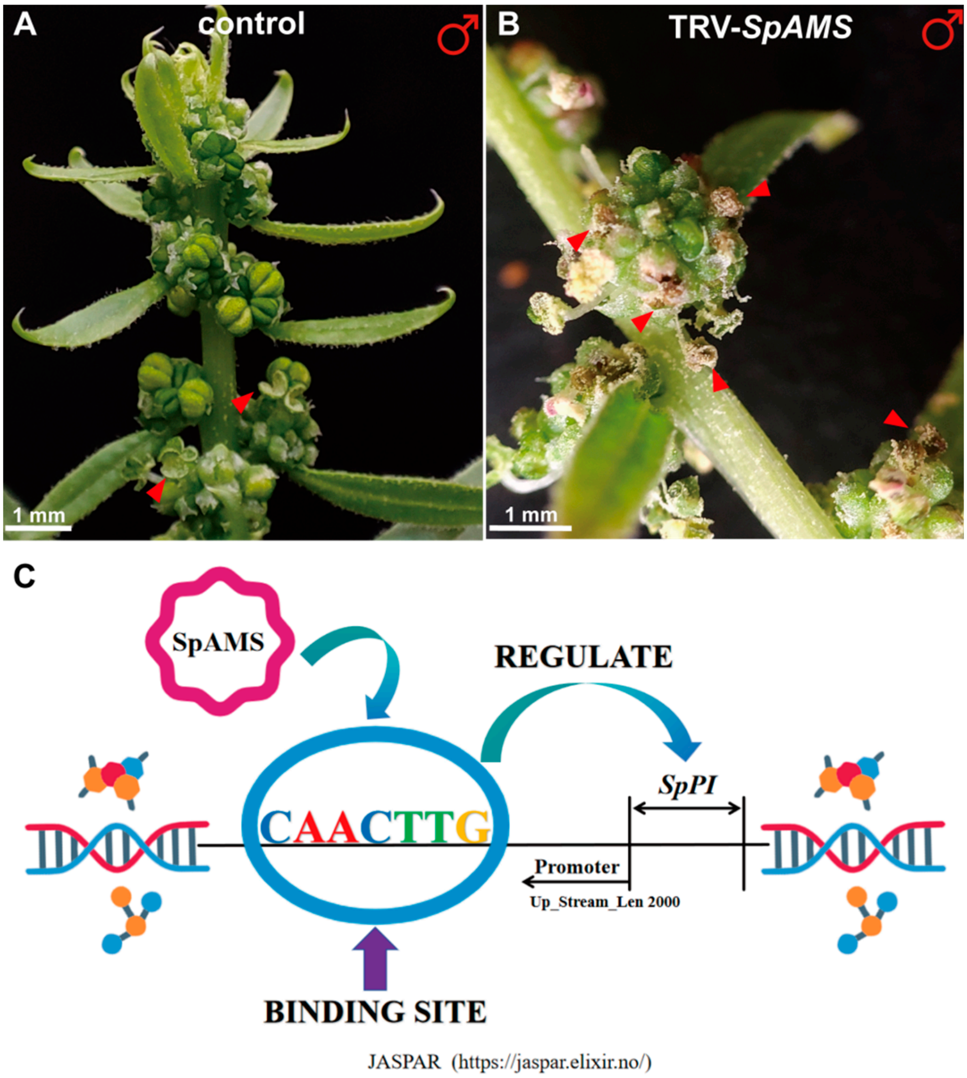

Figure S7. Morphological and Bioinformatics Analysis of *SpAMS* Silencing in Spinach

(A) Normal stamens of untreated Spinach. (B) Withered anthers resulting from VIGS-mediated *SpAMS* knockdown. (C) Transcription-factor binding-site prediction generated with *JASPAR*.

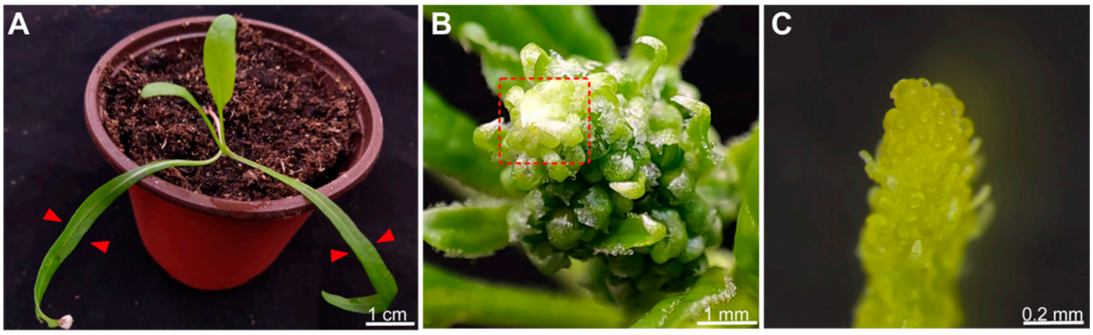

Figure S8. Sampling Sites for VIGS and In Situ Hybridization Experiments

(A) VIGS Infection Site in Spinach. The red arrows indicate the two cotyledons of the spinach seedling. (B) (C) In Situ Hybridization in Anther Tissue. (B) Displays a newly formed spinach anther used for in

situ hybridization experiments. (C) Presents the microscopic structure of the apical meristematic tissue of this anther.

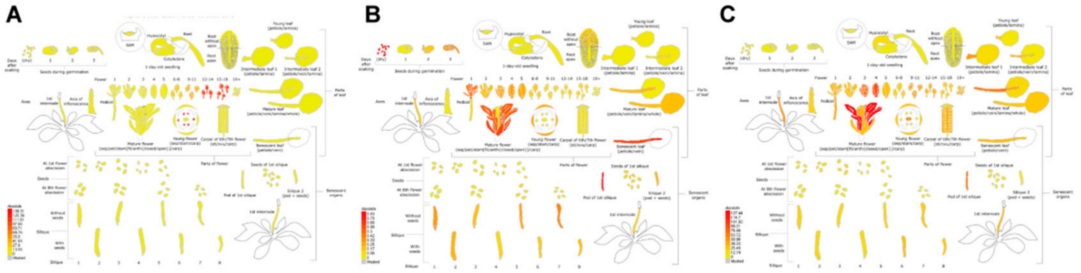

Figure S9. Expression Patterns of *Arabidopsis thaliana* Homologs of *SpAMS* and *SpPGIP*.

(A) Expression sites of *AtAMS*. (B) Expression sites of *AtPGIP1*. (C) Expression sites of *AtPGIP2*.

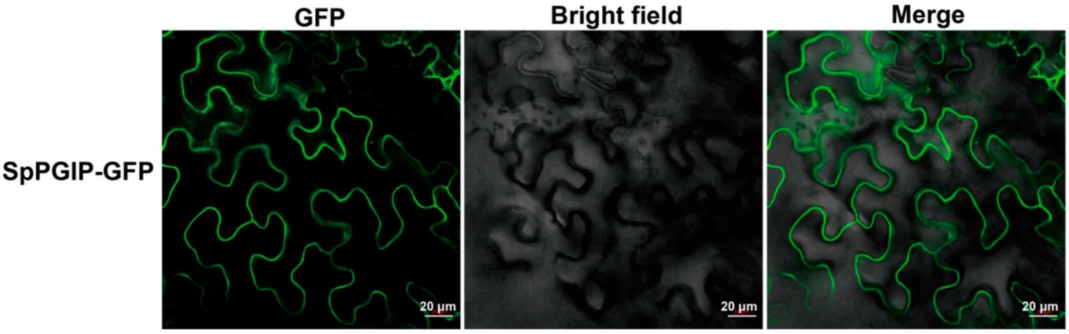

Figure S10. Subcellular localization of SpPGIP-GFP

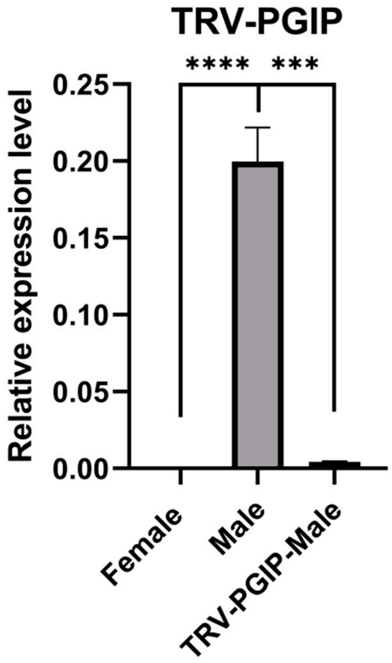

Figure S11. *Expression of SpPGIP in TRV-SpPGIP Plants*

Three biological replicates were conducted utilizing a single sample.****$p<0.001$, ******$p<0.0001$
